# Miiuy Croaker Transferrin Gene and Evidence for Positive Selection Events Reveal Different Evolutionary Patterns

**DOI:** 10.1371/journal.pone.0043936

**Published:** 2012-09-05

**Authors:** Yueyan Sun, Zhihuang Zhu, Rixin Wang, Yuena Sun, Tianjun Xu

**Affiliations:** Laboratory for Marine Living Resources and Molecular Engineering, College of Marine Science, Zhejiang Ocean University, Zhoushan, China; Auburn University, United States of America

## Abstract

Transferrin (TF) is a protein that plays a central role in iron metabolism. This protein is associated with the innate immune system, which is responsible for disease defense responses after bacterial infection. The clear link between TF and the immune defense mechanism has led researchers to consider TF as a candidate gene for disease resistance. In this study, the *Miichthys miiuy* (miiuy croaker) TF gene (MIMI-TF) was cloned and characterized. The gene structure consisted of a coding region of 2070 nucleotides divided into 17 exons, as well as a non-coding region that included 16 introns and spans 6757 nucleotides. The deduced MIMI-TF protein consisted of 689 amino acids that comprised a signal peptide and two lobes (N- and C-lobes). MIMI-TF expression was significantly up-regulated after infection with *Vibrio anguillarum*. A series of model tests implemented in the CODEML program showed that TF underwent a complex evolutionary process. Branch-site models revealed that vertebrate TF was vastly different from that of invertebrates, and that the TF of the ancestors of aquatic and terrestrial organisms underwent different selection pressures. The site models detected 10 positively selected sites in extant TF genes. One site was located in the cleft between the N1 and N2 domains and was expected to affect the capability of TF to bind to or release iron indirectly. In addition, eight sites were found near the TF exterior. Two of these sites, which could have evolved from the competition for iron between pathogenic bacteria and TF, were located in potential pathogen-binding domains. Our results could be used to further investigate the function of TF and the selective mechanisms involved.

## Introduction

As a main transporter in the blood stream of vertebrates, transferrin (TF) is vital to iron metabolism and maintains the iron requirement of cells through its binding and transport of iron [1]. The molecular weights of members of the TF in many phyla range from 70 kDa to 80 kDa [2], [3]. This family which is found in vertebrates and invertebrates, also includes lactotransferrin (LTf, from mammalian milk), melanotransferrin, ovotransferrin (OTf, from bird egg whites) and serum-transferrin (STf, from vertebrate serum) [4]–[6]. The TF protein is responsible for iron transport for storage and the subsequent utilization in metabolic processes, such as DNA synthesis, oxygen and electron transport, cell proliferation and regulation of the immune system. Iron is often a restricting nutrient for bacterial growth; thus, aside from its other functions, TF is considered to be involved in infection resistance and in the control of bacterial development [7]. TF absorbs iron in the gut, between circumjacent sites of storage and usage, maintaining an appropriate iron balance in the body [1], [8], [9]. This protein is also vital to metal transport. The TF gene comprises two functional domains: the N-terminal half domain (N-lobe) and the C-terminal half domain (C-lobe). Each of these two domains consists of ∼335 amino acids, with a highly conserved iron-binding site in each lobe [10], [11]. The N-lobe is apparently more important to iron binding, whereas the C-lobe may be the primary binding site in the TF receptor [12].

Members of the TF family have been found in various species [6], [13], [14]. The TF genes of several species have been cloned and characterized, including those of frog [15], chicken [16], reptiles and several mammals [17]–[19], as well as those of some invertebrates [9], [20]. These genes are also found in several fish species, such as the zebrafish [5], Atlantic cod [21], Atlantic salmon [22], medaka [23], [24] and salmonids [22], [25]–[29], as well as in tetraodons, red seabreams and fugu (data unpublished but may be obtained from the GenBank). The complete nucleotide sequence of the medaka TF gene is ∼8.5 kb in length and organized into 17 exons separated by 16 introns [24]. In addition, molecular evolutionary studies on the transferrins of the salmonid species showed that positive selection for new replacement alleles played an important part during evolution [30] and was responsible for high DNA polymorphism of the TF gene in *Carassius auratus* [31].

Miiuy croaker (*Miichthys miiuy*), an economically important aquaculture species, is extensively farmed in China since the late 1990s and is mainly spread from the western Japan Sea to the East China Sea [32]. Considering its high nutrient content, delicious taste and economic value, the miiuy croaker is considered one of the most important species in the marine industry. Despite its ecological and economic significance, research on its main genes and their involvement in the iron metabolism has been scarcely. In addition, the immune response of fish TF against *Vibrio anguillarum*, which is one of the most menacing bacterial species in aquaculture, remains largely unknown [33].

To date, the TF gene in miiuy croaker, particularly the gene with relevant metabolic and immunologic functions, remains uncharacterized. Hence, this study aims to identify and characterize the TF gene in miiuy croaker. In determining the genomic structure and tissue expression patterns of TF, we hope to offer a clearer understanding of the role of iron-related immune genes of miiuy croaker in its response against a common bacterial pathogen, *V. anguillarum*. Ford [29] found evidence of positive selection in the TF of salmonids. Yang et al. [31] also found evidence of positive selection in multiple antique allelic lineages of TF in the polyploid *C. auratus*. In addition, TF has often been considered a symbol gene in studies on the various genetic aspects of fish [34]. Thus, our particular goals are to find evidence of the evolutionary process underwent by TF genes in the various vertebrate groups during speciation and to investigate the evolutionary mechanisms of the TF genes of aquatic and terrestrial organisms.

## Results and Discussion

### MIMI-TF gene characterization

**Figure 1:**
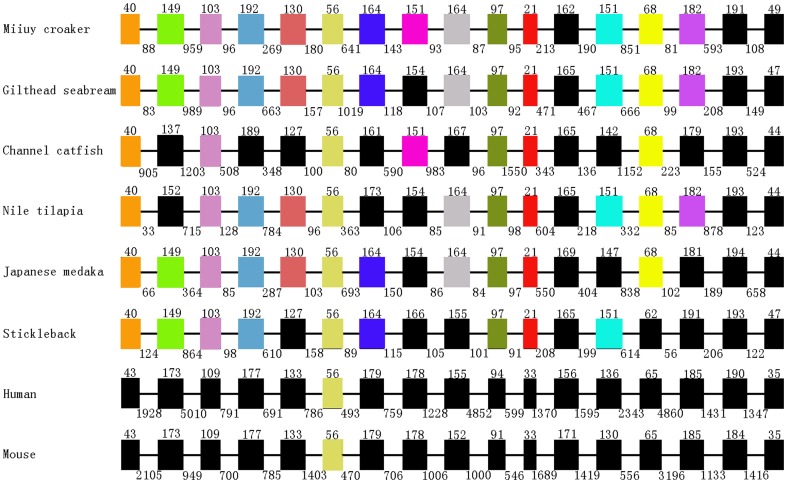
A comparison of the miiuy croaker TF gene structure with the previously published gene structures of other fish species and mammals. Exons were represented by boxes. The length of the exons was indicated on the top of boxes and the length of introns was indicated under the line below each gene structure. And the same color of each column represents that the length of exon was the same.

In this study, the genomic and cDNA sequences of MIMI-TF were cloned and characterized. The genomic fragments showed a MIMI-TF genomic sequence with 6757 nucleotide in length (GenBank accession No. JN969074). The MIMI-TF gene structure was determined from the alignments of the genomic and cDNA sequences. The MIMI-TF genomic DNA sequence described in this study consisted of 17 exons and 16 introns (Fig. 1). The full length MIMI-TF cDNA was 2070 bp (GenBank accession No. JN969073), and encoded a protein of 689 amino acid residues. The N-terminal segment included a high proportion of hydrophobic amino acid residues. The first 18 amino acid residues were predicted using the SMART program (http://www.smart.embl_heidelberg.de/) and were found to consist of two lobes: the N-lobe (24 aa to 336 aa) and C-lobe (339 aa to 679 aa). The Fe-binding residues (Asp-73, Tyr-103, Tyr-200, His-256, Asp-393, Tyr-427, Tyr-522, and His-590) and anion-binding (Thr-128, Lys-132, Thr-452 and Arg-456) residues which were also described by Lambert et al. [13], were conserved in miiuy croaker (Fig. S1). In the N-lobe, Asp-73 was the only amino acid in all eleven TF members as well as among the four iron-binding residues and two anion sites. The amino acids at the other five sites in silver prussian carp, grass carp and zebrafish varied. However, the four iron-binding residues and two anion sites in the C-lobe were all invariable.

A comparison of the TF gene structures of miiuy croaker, gilthead seabream, channel catfish, Nile tilapia, and Japanese medaka, stickleback, human and mouse revealed a highly conserved exon size [35] (Fig. 1). The exons sizes of all vertebrate TF genes showed strong similarities and high conservation. However, this conservation was not observed in the intron sizes, which showed wide variations in all compared species (Fig. 1).

The deduced amino acid sequence of the TF coding region shared a 45.1% to 94% identity with the sequences of previously reported TFs (Table S1). A comparison of the miiuy croaker TF gene structure with the previously published gene structures of other fish species and mammals, showed a high conservation of the exon size (Fig. 1), particularly between those of the miiuy croaker and the gilthead sea bream. These species differed in the length of the amino acid sequence of the deduced protein, with sizes varying from 679 to 698. The deduced miiuy croaker TF protein was composed of 689 amino acids which consisted of an initial peptide and two lobes (N- and C-lobes). The iron- and anion-binding sites of TF among fishes were completely conserved (Fig. S1).

### TF expression in miiuy croaker tissues

**Figure 2:**
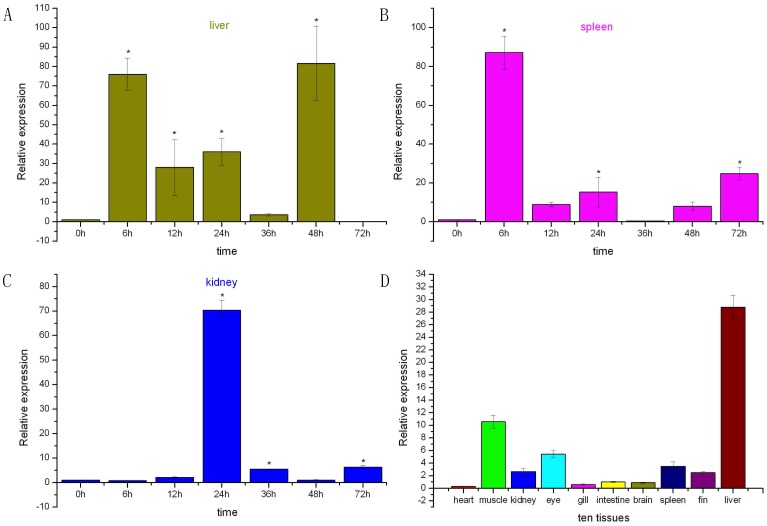
Expression analysis of MIMI-TF by relative quantitative real-time PCR in liver (A), spleen(B) and kidney (C) during 6, 12, 24, 36, 48, and 72 h of induction with *V. anguillarum*, and (D) Expression of TF gene in various tissues (heart, muscle, kidney, eye, gill, intestine, brain, spleen, fin and liver of uninfected miiuy croaker. Deviation bars represented the standard errors (± the SD/SE) of three experiments at each time point.

qRT-PCR was used to study the expression pattern of the TF gene in various tissues. TF expression was observed in ten tested tissues; however, the expression levels in different tissues showed significant differences. TF was highly expressed in the liver and muscle, moderately expressed in the eye, spleen, fin, and kidney, but weakly expressed in the intestine, brain, gill, and heart. The highest expression was detected in the liver, whereas the lowest expression was detected in the heart (Fig. 2).

MIMI-TF expression in the liver, spleen, and kidney was investigated after the challenge experimens with *V. anguillarum*. In the liver, the expression gradually increased after an initial decrease from 0 h to 6 h. The highest expression level was detected at 48 h post-infection, followed by a rapid decrease at 72 h. Moreover, considering the case of pathogens stimulation, it could be understandable that some up-regulation were inevitable to activate downstream signaling molecules, for example, resulting in a great amount of TF synthesis and release. In the kidney, the TF expression was dramatically induced after the challenge tests (Fig. 2). The TF expression in this tissue was stable from 0 h to 12 h. The expression was then sharply up-regulated, reached the peak level at 24 h, and then showed a rapid decrease after 36 h. In the spleen, the expression was constantly up-regulated from the start of infection up to 6 h, and was then sharply down-regulated at 12 h. A slight fluctuation from 12 h to 72 h then followed. However, the expression gradually decreased to a very low level at 36 h (Fig. 2). After induction, the TF gene expression was remarkably up regulated in the liver, spleen and kidney. Time-course analysis showed that the gene was induced at an early time point, and that the signal was strong in the early induction stage. The MIMI-TF gene expression implied the importance in acute immune-related responses against pathogen invasion. This study revealed the vital function of TF during bacterial infection in fish in addition to its role in iron.

### Multiple alignment and phylogenetic analysis

MIMI-TF shared 45% to 94% identity with the TF protein sequences of grass carp, goldfish, zebrafish, common carp, channel catfish, Atlantic cod, Japanese flounder, rainbow trout, medaka, gilthead sea bream, sea bass, and large yellow croaker. The gene also shared a 45% to 46% identity with mammalians (human and mouse) serum TF (Table S1).

**Figure 3:**
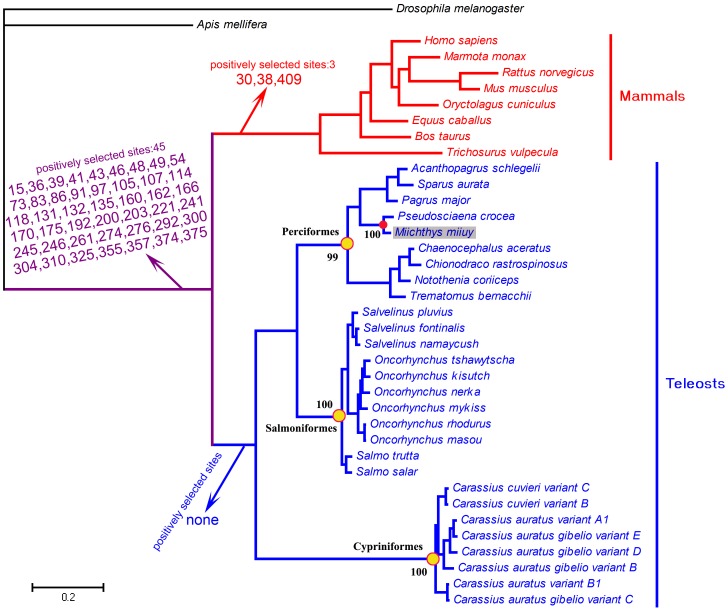
Phylogenetic tree of the nucleotide sequences of transferrin proteins from 38 taxa. The phylogeny of the sequences was estimated using the Bayesian inference implemented in the software MrBayes.

Phylogenetic analysis was performed on the nucleotide sequences from 38 taxa to analyze the evolutionary context of the MIMI-TF gene in the larger context of vertebrates. The teleost TF genes formed three distinct sub-clades: (1) Cypriniformes [posterior probability (PP = 1.00)], (2) Perciformes (PP = 0.99), and (3) Salmoniformes (PP = 1.00). Miiuy croaker was included within the Perciformes subcluster, which had a high PP (PP = 1.00), whereas Cypriniformess and Salmoniformes had their own separate clusters (Fig. 3). The phylogenetic tree showed that the M. miiuy TF was most genetically related to those of *Pseudosciaena crocea* (Fig. 3). This result, together with the highest amino acid sequence identity obtained in the homolog comparison, suggested that the TF gene might have converged into the same function through separate evolutionary paths [36]. The phylogenetic tree constructed by the nucleotide sequences from teleost and mammalian TF genes showed the TF subsets of Perciformes, Salmoniformes and Cypriniformes (different habitat) formed the monophyletic groups, separately (Fig. 3). And the mammals (terrestrial animal) also formed one monophyletic group which was separated with the monophyletic group of fishes (aquatic animal; Fig. 3). We assumed that the different environments might propel TF to evolve in different paths, but eventually they also kept their original function. In addition, most of the fish TF formed their own branches, indicating that the majority of fish TF were generated after the divergence between the species. As an increasing number of teleost TF sequences became available, more meaningful and comprehensive phylogenetic analyses might become feasible and eventually provide a detailed history of TF evolution in low vertebrate.

### Evolutionary process of TF genes

The phylogenetic tree (Fig. 3) was utilized to detect the positive selection in the ancestral lineages of vertebrate, mammalian and teleost genes based on their TF sequences. First, the one-ratio model was compared with the one-ratio (*ω* = 1) model. The results showed that the TF genes were highly conserved and underwent purifying selection (*P*<0.01, Table 1). In addition, Yang [37] pointed out that entire genes with *ω*>1 were generally rare, and that positive selection generally occurred in some structural domains. The free-ratio model was then compared with the one-ratio model. The results showed that each lineage had independent *ω* values (*P*<0.01, Table 1). Lastly, the branch-site models were conducted to determine whether positively selected sites existed in the ancestral lineages of vertebrates (PP = 1.00), mammals (PP = 1.00), and teleosts (PP = 0.79). Positive selection occurred in the ancestral lineages of vertebrates and mammals, but not in teleosts. A site model was then used to detect the possible positively selected sites in the current TF genes of mammals (terrestrial organisms) and teleosts (aquatic organisms). For mammals, the LRT value (2Δln*L*) obtained from the M7–M8 comparison was 11.46, revealing that the model that allowed positive selection was more consistent with the experimental data compared with the model that did not incorporate selection (*P*<0.01, Table 2). Meanwhile, the BEB approach detected one site that underwent positive selection based on the M8 comparison, with BPP values above 0.95 (172, Table 2). On the other hand, M8 was not rejected in the teleosts, and the BEB approach detected nine positively selected sites (BPP>0.95, Table 2). This result might be attributed to the distinct immune function of TF against foreign pathogens. In this function, TF possibly underwent coevolution with the microbial ligands.

**Table 1:** Likelihood ratio tests (LRT) of branch-models and branch-site models for transferrin (TF) genes.

Model	Np^1^	Ln likelihood	Parameter estimates	Model compare	Positive selection sites	2Δln*L* 2 (*p*-value)
**Site-model**						
A: One-ratio	75	18792.50	*ω* = 0.304		None	
B: Omega = 1	74	19220.65	*ω* = 1.0	B vs A		856.30(*P* = 0)
C: Free-ratio	147	18597.46	variable *ω* by branch	C vs A	n/a	390.07(*P* = 0)
**Branch-site model**						
1: Null-Ver^3^	77	18335.40				
2: Ver	78	18317.44		1 vs 2	15, 36, 39, 41, 43, 46, 48, 49, 54, 73, 83, 86, 91, 97, 105, 107, 114, 118, 131, 132, 135, 160, 162, 166, 170, 175, 192, 200, 203, 221, 241, 245, 246, 261, 274, 276, 292, 300, 304, 310, 325, 355, 357, 374, 375	35.94(*P*<0.001)
3: Null-Mam^4^	77	18348.28				
4: Mam	78	18338.35		3 vs 4	30, 38, 409	19.85(*P*<0.001)
5: Null-Tel^5^	77	18351.44				
6:Tel	78	18341.75		5 vs 6	not found	19.37(*P*<0.001)

1 Number of parameters.

2 Twice the difference of ln [likelihood] between the two models compared.

3 Ver = Ancestor branch of the vertebrates examined in present study.

4 Mam = Ancestor branch of the mammals examined in present study.

5 Tel = Ancestor branch of the Teleosts examined in present study.

**Table 2:** Site model tests on TF genes in the mammalian, fish, and Perciformes subsets.

Model	Np^a^	Parameter estimates	Ln likelihood	Compared models	Positive selection sites^b^	2Δln*L* (*p*-value)
**Data set: mammalian**						
M0: one-ratio	16	*ω* = 0.275	−4525.92			
M3: discrete	20	*ω_0_* = 0.034, *p_0_* = 0.498, *ω_1_* = 0.475, *p_1_* = 0.417, ***ω_2_*** ** = 1.860**, *p_2_* = 0.086	−4395.08	M3 vs M0	Not analyzed	261.69(*P = *0)
M1a: nearly neutral	17	*ω_0_* = 0.085, *p_0_* = 0.653, *ω_1_* = 1.000, *p_1_* = 0.347	−4408.75			
M2a: positive selection	19	*ω_0_* = 0.086, *p_0_* = 0.645, *ω_1_* = 1.000, *p_1_* = 0.331, ***ω_2_*** ** = 3.047**, *p_2_* = 0.024	−4406.85	M2 vs M1	Not found	3.79(*P* = 0.150)
M7: β	17	*p* = 0.299, *q* = 0.628	−4401.61			
M8: β and ω	19	*p_0_* = 0.940, *p_1_* = 0.060, ***ω*** ** = 2.082**,*p* = 0.377, *q* = 1.012	−4395.88	M8 vs M7	172	11.46(*P* = 0.003)
**Data set: teleosts**						
M0: one-ratio	56	*ω* = 0.465	−12655.98			
M3: discrete	60	*ω_0_* = 0.062, *p_0_* = 0.046, *ω_1_* = 0.685, *p_1_* = 0.438, ***ω_2_*** ** = 2.351**, *p_2_* = 0.101	−12270.80	M3 vs M0	Not analyzed	770.35(*P* = 0)
M1a: nearly neutral	57	*ω_0_* = 0.101, *p_0_* = 0.570, *ω_1_* = 1.000, *p_1_* = 0.430	−12312.08			
M2a: positive selection	59	*ω_0_* = 0.101, *p_0_* = 0.545, *ω_1_* = 1.000, *p_1_* = 0.405, ***ω_2_*** ** = 3.242**, *p_2_* = 0.050	−12277.57	M2 vs M1	65, 128, 219, 294, 303, 406, 425, 514	69.02(*P* = 0)
M7: β	57	*p* = 0.304, *q* = 0.400	−12309.14			
M8: β and ω	59	*p_0_* = 0.925, *p_1_* = 0.075, ***ω*** ** = 2.543**,*p* = 0.354, *q* = 0.543	−12268.35	M8 vs M7	65, 98, 128, 219, 294, 303, 406, 425, 514	81.58(*P* = 0)

a Number of parameters.

b Only positively selected sites with a posterior probability equal or greater than 95% were indicated by the Bayes Empirical Bayes (BEB) approach.

From the above results, our data could be reasonably interpreted based on the evolutionary relationships in the phylogenetic tree. We found 45 positively selected sites in the ancestral lineages of vertebrates, this number was significantly higher than that found in other ancestral lineages of animals or in the subset of the current species (Table 1; Fig. 3). The results indicated that the TF in the vertebrate ancestors suffered a wider range of selected pressure compared with others. TF was an iron-binding protein that was involved the selective delivery of iron to tissues as well as in disease defense responses [38], [39]. The positive selection in the vertebrate ancestral lineage might be associated with oxygen transport, energy requirement, or immune function of vertebrates. Given that vertebrates possess more complex respiratory, circulatory, metabolic, immune, and motor systems than invertebrates, they might require a higher amount of TF for defense against pathogens or for iron transport which was an integral part of the oxyhemoglobin molecule that delivered oxygen to tissues [38]. Given the considerable differences between the biological structure and physiology functions of invertebrates and vertebrates, the vertebrates might have evolved a large number of sites in their functional genes to perfect their own structures and protein functions. Meanwhile, the results revealed the occurrence of positive selection in the ancestral lineage of mammals but not in teleosts, indicating that the ancestral lineages of mammals and teleosts underwent different selected pressures. The ancestors of terrestrial animals might have evolved further to adapt to terrestrial environments, particularly landed with pathogen and oxygen levels that significantly differ from those of the ocean. On the other hand, the ancestors of teleosts might have still remained in waters whose conditions were similar to those of their previous environment. Hence, they were able to adapt to the environment without changing the nature of their functional genes. These results indicated that the ancestors of aquatic and terrestrial organisms might have followed different evolutionary pathways. The site models found positively selected sites in the current TF sequences of mammals and teleosts. We theorize that as the years passed, the oceans and lands all experienced significant changes. This phenomenon, led the current aquatic and terrestrial organisms to adapt to their new environment, evolving their functional proteins.

**Figure 4:**
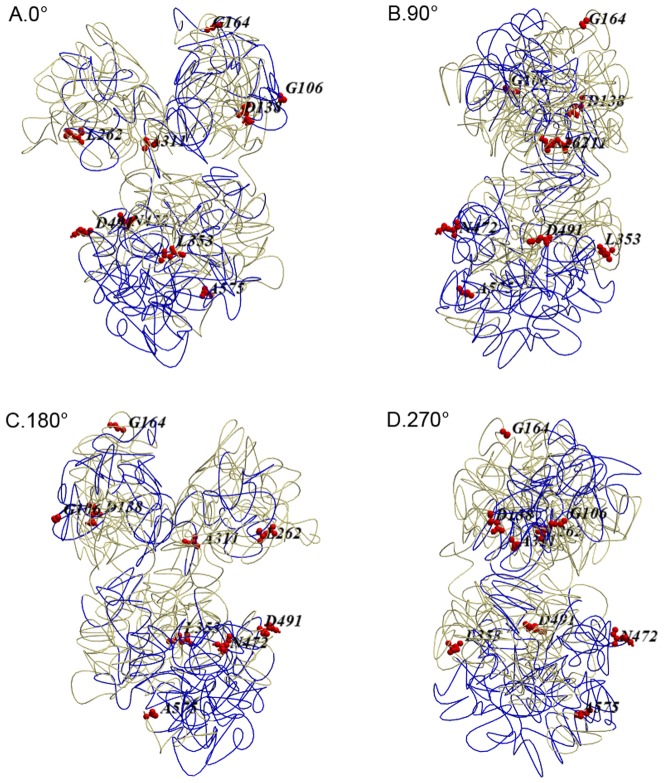
Three-dimensional structure of human transferrin proteins. Images depicted represent four different perspectives (the angles of view are 0°, 90°, 180°, and 270°for the front, back and side via rotation in 90°increments around the vertical axis) of the surface of a space-filled model of human structure.

Ford [29] proposed that the general three-dimensional structure of TF was likely conserved among diverse species. Therefore, the known crystal structure of human TF was used to analyze our data [40]. A total of 10 positively selected sites were found in the extant vertebrate TF (Table 2). A comparison of the TF under study with the human TF showed similarities in their structures, particularly in the N- and C-lobes. Retzer et al. [40] identified the human TF sequences bound by the pathogenic bacteria TF-binding protein B (TbpB). These binding domains were shaded blue in Figure 4, whereas the positively selected sites detected in the extant sequences, which could be homologous to the human TF structure, were shown as red balls [40]. Only one of the ten sites identified as positively selected did not have sites homologous to the human TF sequence. One of the remaining sites was located in the cleft between the N1 and N2 domains, and was expected to bond directly to iron or an anion through a hydrogen bond [31] (A311; Fig. 4). The variation in the site might indirectly affect the capability of TF to bind to or release iron [31]. The other eight remaining sites were found near the TF exterior, away from the interdomain cleft responsible for ions binding. However, these sites were all located on the TF surface. This finding was consistent with the bacterial-selection hypothesis [29]. Two sites were located in the potential pathogen-binding domains; these sites might have evolved from the competition for iron between pathogenic bacteria and TF [29] (G106, N472; Fig. 4). The variations in these outer sites might result in the opening or closing of interdomain clefts or half molecules to different extents and ultimately might affect the binding or release of iron [41]. Our findings could be used to further investigate the functions of TF and the selective mechanisms involved.

## Materials and Methods

### Ethics statement

This study has been approved by the permission (ZJOU-AWC-12-05) from the Zhejiang Ocean University Animal Welfare Committee.

### Fish sampling and challenge experiments

Healthy miiuy croakers (800±25 g) were collected from Zhoushan Fisheries Research Institute (Zhejiang, China). The fishes were acclimatized in a recirculating seawater system at ambient temperature and under a natural photoperiod for at least one week. The fishes were fed twice daily with commercial pellets until satiety.

Miiuy croaker challenge experiments with *V. anguillarum* were performed as previously described [42], the miiuy croaker was injected with 1 mL bacteria *V. anguillarum* suspension, which was made after *V. anguillarum* centrifuge to approximately 3×10^7^ CFU/ml in phosphate-buffered saline. And uninfected fish were maintained in separate tanks as control group. Infected fish were respectively killed at 6, 12, 24, 36, 48 and 72 h after injection treatment. Tissues (liver, spleen and kidney) were removed and then stored at −70°C until use. To determine the TF gene expression in the healthy miiuy croakers, ten tissue samples from the heart, muscle, kidney, eye, gill, intestine, brain, spleen, fin and liver were collected from uninfected miiuy croakers and placed in tubes. The samples were then immediately frozen in liquid nitrogen after dissection and then separately stored at −80°C prior to RNA extraction.

### DNA and RNA extraction, cDNA synthesis

Genomic DNA was extracted from the fin samples of miiuy croakers via the previously described standard phenol-chloroform method [43]. The DNA samples were then stored at −20°C prior to polymerase chain reaction (PCR) analysis. Total RNA was extracted from the different tissues of adult individuals using Trizol reagent (Qiagen) in accordance with the manufacturer's instructions. cDNA synthesis was performed using a QuantScript RT Kit (TIANGEN) according to the manufacturer's protocol, the cDNAs were then stored at −20°C for later use.

### Primer design, PCR amplification and cloning

To obtain the full length cDNA sequence, two pairs of primers (HM-transferrin-Gap1-1F/1R and HM-transferrin-Gap2-1F/1R) were designed according to a MIMI-TF partial cDNA sequence. This partial sequence was obtained in our laboratory from the spleen cDNA library of miiuy croaker via expressed sequence tag (EST) analysis [44]. In addition, two pairs of primers (Transferrin-RT-F/R and β-actin-RT-F/R) were designed and subsequently used in the study of the MIMI-TF gene expression. To identify the genomic organization of TF, three primers pairs (HM-transferrin-intron-1F/1R, HM-transferrin-intron-2F/2R and HM-transferrin-intron-3F/3R) were designed to amplify the introns from the miiuy croaker genome (Table S2). The final volume (25 µl) of the PCR mixture contained 0.2 µmol of each primer, 2.5 µl of 10×PCR buffer, 0.2 mmol dNTP, 1 unit Taq polymerase (TaKaRa) and 1 µl cDNA/DNA template. The PCR reactions were performed under the following conditions: pre-denaturation at 94°C for 3 min; 35 cycles of denaturation at 94°C for 30 s, annealing at 55°C for 30 s, extension at 72°C for 2 min; and a final extension at 72°C for 10 min. The PCR products were then cloned into PBS-T vector (TIANGEN). At least three clones were sequenced by the M13F/R sequencing primers.

### Analysis of MIMI-TF expression

The mRNA expression patterns of transferrin gene in different tissues (heart, muscle, kidney, eye, gill, intestine, brain, spleen, fin and liver) of healthy miiuy croakers and in three tissues (liver, spleen and kidney) of infected and health miiuy croakers were determined using real-time RT-PCR. Tissue samples from three individuals were mixed for RNA preparation. To reduce the possibility of genomic DNA amplification, the Transferrin-RT-F/R primer sequence was designed to span the border between exon13 and exon15. The Transferrin-RT-F/R was used to amplify the miiuy croaker TF gene fragment (Table S2). Real-time quantitative PCR (qRT-PCR) was conducted on a 7500 real-time PCR system (Applied Biosystems, USA) using the SYBRR® *premix ExTaq*™ Kit (TaKaRa). The reaction conducted without the template was used as the blank control. PCR amplification was performed in triplicate wells and the cycling conditions were as follows: 10 s at 95°C, followed by 40 cycles at 95°C for 5 s and at 60°C for 34 s. Dissociation curve analysis was performed after each assay to determine the target specificity. The β-actin expression was used as the internal control for the MIMI-TF gene expression analysis. The β-actin-RT-F/R primer (Table S1) was used for the RT-PCR of β-actin expression. Fluorescent detection was performed after each extension step. The dissociation curve was analyzed after thermocycling to determine whether a specific-sized single amplicon was amplified. MIMI-TF expression was determined via the 2^−ΔΔC^T method [45] and subjected to a one-way analysis of variance (one-way ANOVA) using SPSS software.

### Sequences analysis, alignments and phylogenetic analysis

All the TF cDNA sequences used in this study were obtained from the GenBank (http://www.ncbi.nlm.nih.gov/Genbank/) and Ensemble (http://www.ensembl.org/) databases: eight TF sequences from eight mammals and twenty-eight sequences from twenty-three teleosts, together with two TF from two invertebrates as outgroups. Taxonomic information and accession numbers are provided in Table S3. The SignalP 4.0 Server was adopted for signal peptide prediction [46]. The MUSCLE software was used for the alignment of the putative amino acid sequences of miiuy croaker and other known vertebrates using default alignment parameters [47]. The sequence identities were determined using the MEGALIGN program of DNASTAR [48].

The jModeltest software was used to select the optimal substitution model for our collected nucleotides data [49]. The Akaike information criterion (AIC) was used to choose the optimal model. A phylogenetic tree was constructed via the Bayesian approach using MrBayes v3.1.2 [50]; the program was run using 10,000,000 generations under GTR+I+G model, with 25% of trees burned. The resulting phylogenetic tree was then visualized and edited using TreeView [51].

### Evolutionary analysis

The phylogenetic tree (Fig. 3) was used to determine the possible effects of diverse environments on the selective pressures on the ancestors of aquatic or terrestrial organisms. The selective pressures at the ancestral branches of vertebrates, mammals and teleosts were analyzed using the maximum likelihood (ML) methods in the CODEML program of PAML v4 [52]. First, the one-ratio model which assumed that all branches have only one ratio, was used to identify the selective pressures in all TF genes. Second, the free ratio that allowed varied *ω* ratios in every branch was used to determine via the likelihood ratio test (LRT) whether this model suitably fitted the data compared with the one-ratio model. The branch-site model was then used to identify the ancestral foreground lineages of interest of vertebrates, mammals and teleosts.

The subsets of mammalian and teleost TF sequences, both of which used the TF of the two invertebrates as the out group, were used to account for the different functional and structural constraints experienced by individual site–domains in the site model [45]. Six site models were used on the teleost and mammalian TF sequences subsets to investigate the possible positively selected sites among those lineages. In all cases, the two-fold differences of log-likelihood values (2Δln*L*) between each of the two nested models were calculated following a chi-square distribution, with the degrees of freedom equal to difference in the number of parameters between the nested models [52]. The Bayes empirical Bayes (BEB) were used in the M2a and M8 models to calculate the Bayesian posterior probability (BPP) of the codon sites under a positive selection [53].

### Structure prediction

Three-dimensional models of TF were constructed using human template models (PDB code: 3QYT), following the homology modeling procedure described in a previous paper [39]. The resulting theoretical model was displayed and analyzed using RasMol 2.7.2 [54].

## Supporting Information

Figure S1 **Multiple alignments of amino acid sequences of miiuy croaker transferrin with those of other fish transferrin genes.** Multiple alignments were performed with Muscle (provided EMBLEBI (http://www.ebi.ac.uk/Tools/muscle/index.html). The region of N-lobe and C-lobe, which was predicted by SMART program (http://www.smart.embl-heidelberg.de/) Anion- and iron-binding residues of each lobe of transferrin were marked by red color and green color, respectively. Ol: *Oryzias latipes* [Gene Bank ID: BAF81983]; Ip: *Ictalurus punctatus* [GenBank ID: FJ176740]; Cg: *Carassius gibelio* [GenBank ID: AAK92216]; Ci: *Ctenopharyngodon idella* [GenBank ID:AAR20997]; Dr: *Danio rerio* [GenBank ID: DAA01798]; Sa: *Sparus aurata* [GenBank ID: JF309046]; As: *Acanthopagrus schlegelii* [Gen Bank ID: AAQ63949]; Pm: *Pagrus major* [GenBank ID: AAP94279]; On: *Oreochromis niloticus* [GenBank ID: ABB70391]; Ok: *Oncorhynchus kisutch* [GenBank ID: BAA13759].
(JPG)

Table S1 **Transferrin amino acid identity determined by the DNA STAR.**
(DOC)

Table S2 **Primers used in this study.**
(DOC)

Table S3 **Organisms and accession numbers of the transferrin cDNA sequences used in this paper.**
(DOC)
