# Supplementary figures and images for: Miiuy Croaker Transferrin Gene and Evidence for Positive Selection Events Reveal Different Evolutionary Patterns
Source: PLoS One. 2012 Sep 5;7(9):e43936. doi: 10.1371/journal.pone.0043936 (PMC3434209; doi:10.1371/journal.pone.0043936)

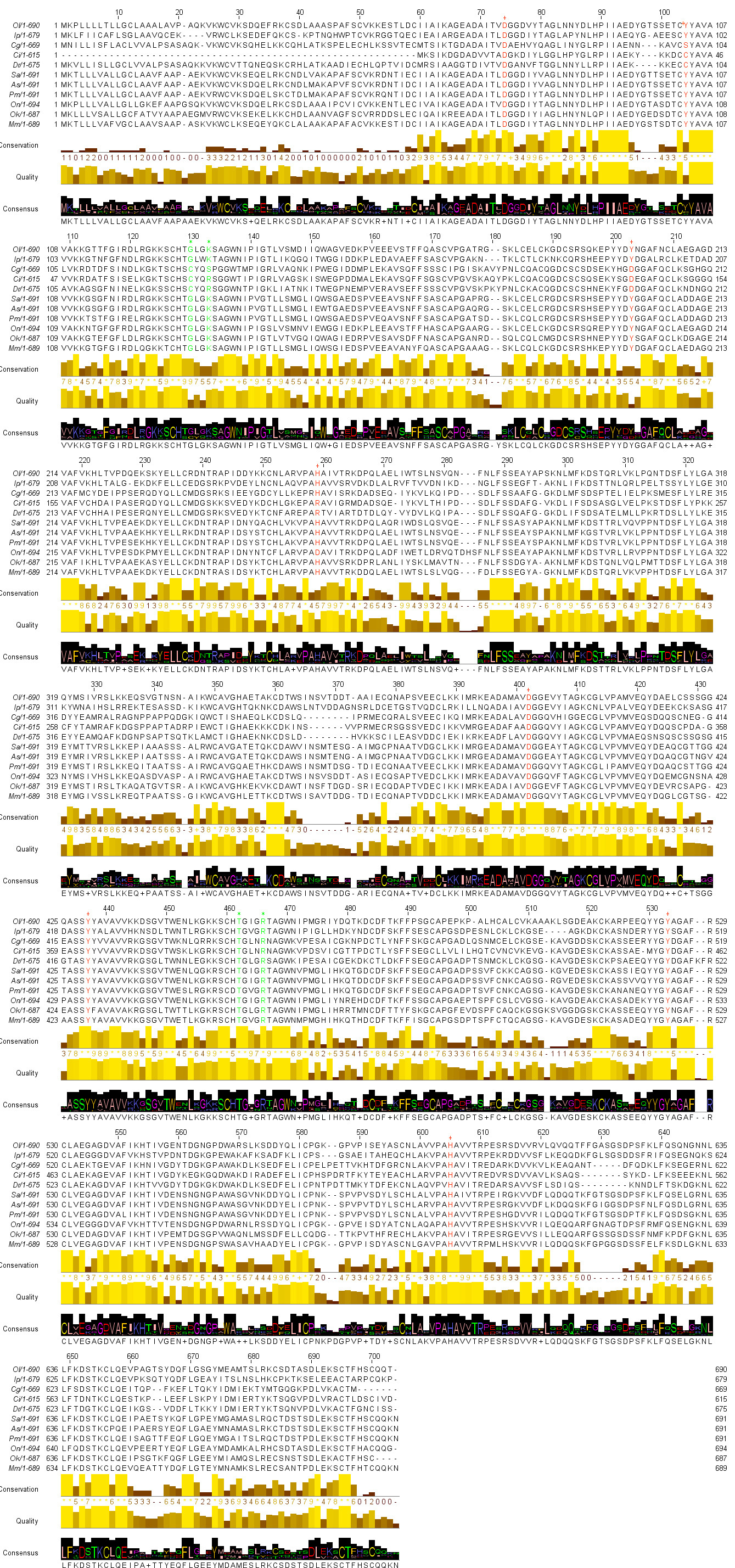

Supplement: Figure S1 — Multiple alignments of amino acid sequences of miiuy croaker transferrin with those of other fish transferrin genes. Multiple alignments were performed with Muscle (provided EMBLEBI (http://www.ebi.ac.uk/Tools/muscle/index.html). The region of N-lobe and C-lobe, which was predicted by SMART program (http://www.smart.embl-heidelberg.de/) Anion- and iron-binding residues of each lobe of transferrin were marked by red color and green color, respectively. Ol: Oryzias latipes [Gene Bank ID: BAF81983]; Ip: Ictalurus punctatus [GenBank ID: FJ176740]; Cg: Carassius gibelio [GenBank ID: AAK92216]; Ci: Ctenopharyngodon idella [GenBank ID:AAR20997]; Dr: Danio rerio [GenBank ID: DAA01798]; Sa: Sparus aurata [GenBank ID: JF309046]; As: Acanthopagrus schlegelii [Gen Bank ID: AAQ63949]; Pm: Pagrus major [GenBank ID: AAP94279]; On: Oreochromis niloticus [GenBank ID: ABB70391]; Ok: Oncorhynchus kisutch [GenBank ID: BAA13759]. (JPG) [file pone.0043936.s001.jpg]
