# Supplementary material for: Miiuy Croaker Transferrin Gene and Evidence for Positive Selection Events Reveal Different Evolutionary Patterns
Source: PLoS One. 2012 Sep 5;7(9):e43936. doi: 10.1371/journal.pone.0043936 (PMC3434209; doi:10.1371/journal.pone.0043936)
Supplement: Table S1 — Transferrin amino acid identity determined by the DNA STAR. (DOC) [file pone.0043936.s002.doc]

**Table S1** Primers used in this study.

| Primer name | Primer sequences(5’ to 3’) |
| --- | --- |
| HM-transferrin-Gap1-1F | CCCGCCGTCCAGCATGAA |
| HM-transferrin-Gap1-1R | CAGACTTCCCCAACCCA |
| HM-transferrin-Gap2-1F | TGAAGAAAGAAAGCACCATC |
| HM-transferrin-Gap2-1R | AAGCCACTTGTCCAGCAT |
| HM-transferrin-intron-1F | CCTAGTGGCGTTTGTTGG |
| HM-transferrin-intron-1R | CCTTGTGGGACCTGGAAC |
| HM-transferrin-intron2-2F | AGCAGCAGGCAGCAAACT |
| HM-transferrin-intron2-2R | CTTCTTCAGGCAATCGTCA |
| HM-transferrin-intron-3F | CTTACTATGCTGTCGCTGTG |
| HM-transferrin-intron-3R | AAAGTGCAAGATTTCTCCA |
| HM-transferrin-intron4-1F | ACATGGGCATTGTCAGTT |
| HM-transferrin-intron4-1R | CTAAAGGCTCCAGCGTAG |
| Transferrin-RT-F | GCTACGCTGGAGCCTTTA |
| Transferrin-RT-R | TTGGCACTGGACCCTTAC |
| β-actin-RT-F | GTGATGAAGCCCAGAGCA |
| β-actin-RT-R | CGACCAGAGGCATACAGG |
