# Supplementary material for: Miiuy Croaker Transferrin Gene and Evidence for Positive Selection Events Reveal Different Evolutionary Patterns
Source: PLoS One. 2012 Sep 5;7(9):e43936. doi: 10.1371/journal.pone.0043936 (PMC3434209; doi:10.1371/journal.pone.0043936)
Supplement: Table S2 — Primers used in this study. (DOC) [file pone.0043936.s003.doc]

**Table S2** Organisms and accession numbers of the transferrin cDNA sequences used in this paper.

| Species | Gene name | Accession no. |
| --- | --- | --- |
| Invertebrate |  |  |
| *Drosophila melanogaster* | TF | AF061268.1 |
| *Apis mellifera* | TF | AY336528.1 |
| Vertebrate |  |  |
| Mammalia |  |  |
| Primates |  |  |
| *Homo sapiens* | TF | S95936 |
| *Mus musculus* | TF | BC092046.1 |
| *Rattus norvegicus* | TF | X77158.1 |
| *Oryctolagus coniculus* | TF | X58533.1 |
| *Trichosurus vulpecula* | TF | Af092510 |
| *Marmota monax* | TF | AY288100.1 |
| Perissodactyla |  |  |
| *Equus caballus* | TF | M69020.1 |
| *Bos taurus* | TF | U02564.1 |
| Perciformes |  |  |
| *Notothenia coriiceps* | TF | AM419437.1 |
| *Tremomus bernahii* | TF | AM419436.1 |
| *Chaenocephalus aceratus* | TF | AM419435.1 |
| *Chionodro rastrospinosus* | TF | AM419434.1 |
| *Sparus aurata* | TF | JF309047.1 |
| *Pagrus major* | TF | AY335444.1 |
| *Acanthopagrus schlegelii* | TF | AY365052.1 |
| *Pseudosciaena crocea* | TF | AM709639.1 |
| *Miichthys miiuy* | TF | JN969073 |
| Salmoniformes |  |  |
| *Salmo salar* | TF | L20313.1 |
| *Salmo trutta* | TF | D89091.1 |
| *Salvelinus pluvius* | TF | D89088.1 |
| *Salvelinus namaycush* | TF | D89090.1 |
| *Salvelinus fontinalis* | TF | D89089.1 |
| *Oncorhynchus masou* | TF | D89087.2 |
| *Oncorhynchus rhodurus* | TF | D89086.2 |
| *Oncorhynchus kisutch* | TF | D89084.1 |
| *Oncorhynchus mykiss* | TF | D89083.1 |
| *Oncorhynchus tshawytscha* | TF | AH008271.2 |
| *Oncorhynchus nerka* | TF | D89085.1 |
| Cypriniformes |  |  |
| *Carassius cuvieri variant C* | TF | AY323918.1 |
| *Carassius cuvieri variant B* | TF | AY323917.1 |
| *Carassius auratus variant B1* | TF | AF518747.1 |
| *Carassius auratus variant A1* | TF | AF518746.1 |
| *Carassius auratus gibelio variant E* | TF | AF518745.1 |
| *Carassius auratus gibelio variant D* | TF | AF518744.1 |
| *Carassius auratus gibelio variant C* | TF | AF457151.1 |
| *Carassius auratus gibelio variant B* | TF | AF457150.1 |

Note TF = transferrin.
