# Supplementary material for: Miiuy Croaker Transferrin Gene and Evidence for Positive Selection Events Reveal Different Evolutionary Patterns
Source: PLoS One. 2012 Sep 5;7(9):e43936. doi: 10.1371/journal.pone.0043936 (PMC3434209; doi:10.1371/journal.pone.0043936)
Supplement: Table S3 — Organisms and accession numbers of the transferrin cDNA sequences used in this paper. (DOC) [file pone.0043936.s004.doc]

**Table S3** Transferrin amino acid identity determined by the DNASTAR.

| Species | English name | Accession number | Miiuy croaker |
| --- | --- | --- | --- |
|  |  |  | Identity(%) |
| *Larimichthys crocea* | Great yellow croaker | CAM96032.1 | 94.0 |
| *Dicentrarchus labrax* | Sea bass | ACN80997.1 | 80.7 |
| *Sparus aurata* | Gilthead seabream | AEA41140.1 | 78.4 |
| *Paralichthys olivaceus* | Japanese flounder | AAF33234.1 | 64.3 |
| *Oryzias latipes* | Medaka | BAA10901.1 | 76.2 |
| *Cyprinus carpio* | Common carp | AF57604.1 | 50.5 |
| *Ictalurus punctatus* | Channel catfish | NP_001187249.1 | 59.2 |
| *Ctenopharyngodon idella* | Grass carp | AAR20997.2 | 45.1 |
| *Gadus morhua* | Atlantic cod | AAB08440.1 | 60.6 |
| *Oncorhynchus mykiss* | Rainbow trout | NP_001118024.1 | 70.1 |
| *Carassius auratus variant B1* | Goldfish | AF518747_1 | 48.6 |
| *Danio rerio* | Zebrafish | DAA01798.1 | 50.0 |
| *Homo sapiens* | Human | AAB22049.1 | 46.6 |
| *Mus musculus* | Mouse | NP_598738.1 | 45.7 |
